# A YOLO-V5 approach for the evaluation of normal fillings and overhanging fillings: an artificial intelligence study

**DOI:** 10.1590/1807-3107bor-2024.vol38.0098

**Published:** 2024-09-30

**Authors:** Nilgün AKGÜL, Cemile YILMAZ, Elif BILGIR, Özer ÇELIK, Oğuzhan BAYDAR, İbrahim Şevki BAYRAKDAR

**Affiliations:** (a) Pamukkale University, Faculty of Dentistry, 1Department of Restorative Dentistry, Denizli, Türkiye; (b) Afyonkarahisar Health Science University, Faculty of Dentistry, Department of Restorative Dentistry, Afyonkarahisar, Türkiye; (c) Eskisehir Osmangazi University, Faculty of Dentistry, Department of Oral and Maxillofacial Radiology, Eskişehir, Türkiye; (d) Eskisehir Osmangazi University, Faculty of Science, Department of Mathematics-Computer, Eskisehir, Türkiye

**Keywords:** Artifical Intelligence, Radiography, Panoramic, Deep Learning, Dentistry

## Abstract

Dental fillings, frequently used in dentistry to address various dental tissue issues, may pose problems when not aligned with the anatomical contours and physiology of dental and periodontal tissues. Our study aims to detect the prevalence and distribution of normal and overhanging filling restorations using a deep CNN architecture trained through supervised learning, on panoramic radiography images. A total of 10480 fillings and 2491 overhanging fillings were labeled using CranioCatch software from 2473 and 1850 images, respectively. After the data obtaining phase, validation (80%), training 10%), and test-groups (10%) were formed from images for both labelling. The YOLOv5x architecture was used to develop the AI model. The model’s performance was assessed through a confusion matrix and sensitivity, precision, and F1 score values of the model were calculated. For filling, sensitivity is 0.95, precision is 0.97, and F1 score is 0.96; for overhanging were determined to be 0.86, 0.89, and 0.87, respectively. The results demonstrate the capacity of the YOLOv5 algorithm to segment dental radiographs efficiently and accurately and demonstrate proficiency in detecting and distinguishing between normal and overhanging filling restorations.

## Introduction

Dental fillings, a common procedure for treating cavities and restoring damaged teeth, can lead to complications if overhangs occur. Overhang is a situation in which a restorative material protrudes beyond the limits of the cavity preparation. This is considered a type of iatrogenic error in terms of the anatomical form of the restoration. This issue can be caused by faulty restorative methods and morphological variations in the cervical area of the tooth, such as concavities, fluting, and furcation. Such variations make it challenging to accurately adapt the matrix band and wedge to the gingival cavity margin, resulting in an ill-fitting restoration with overhang.^1^ To prevent overhanging fillings and associated complications, dentists must ensure precise placement and contouring of the filling material.^1-3^ However, studies indicate a significant prevalence of overhanging dental restorations (ODRs), ranging from 25% to 76% of restored tooth surfacess.^4,5^

Given the transformative potential of artificial intelligence (AI) in dentistry, this study focuses on the role of deep learning, specifically deep convolutional neural networks (CNNs). AI in dentistry uses machine learning algorithms, computer vision, and natural language processing to improve diagnostics and treatment planning, providing opportunities for better patient outcomes and increased efficiency.

AI is a rapidly evolving field that has the potential to transform many aspects of healthcare, including dentistry. AI in dentistry involves the use of machine learning algorithms, computer vision, natural language processing, and other AI technologies to improve diagnostic and treatment options, enhance patient outcomes, and increase efficiency. AI can be used in many areas, such as image analysis, treatment planning, patient communication, robotic dentistry, and predictive analytics in dentistry.^6,7^ AI algorithms can analyze radiographic images to detect and diagnose conditions such as dental caries, periodontal disease, and oral cancer.^8^ Deep learning, a subfield of AI, has attracted much attention in recent years due to its rapid development. Among the various deep learning models, deep CNNs have been extensively studied. They offer excellent performance in analyzing image data, including detection, classification, quantification, and segmentation. This is due to the development of self-learning algorithms and advances in computing power.^9^ Deep learning is being explored in the dental field to identify and analyze various anatomical variables such as orthodontic landmarks, dental caries, periodontal disease, and osteoporosis. However, these applications are still in the early stages of development.^7,10,11^

The current study aims to assess the performance and efficacy of deep learning algorithms, particularly the YOLOv5x architecture, in identifying both normal and overhanging dental fillings using performance metrics. The study is framed around hypotheses to clarify whether these algorithms can effectively detect normal and overhanging dental fillings with high sensitivity and accuracy (H_1_) or not (H_0_). Given the critical need for precise, fast, and sensitive detection in dental image analysis, our research aims to contribute to the advancement of automated dental diagnostic systems.

## Methods

### Ethical approval and study design

**Figure 1:**
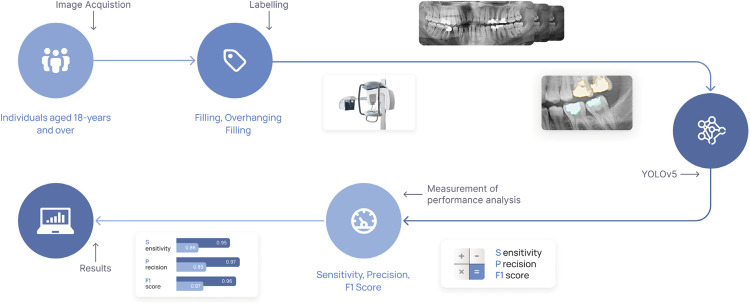
Schematic representation of the study design

The study protocol was authorized by the 04.10.2022/22), and it adhered to the Helsinki Declaration’s standards (NCT06022731). In our study, a YOLOV5 model implemented in Pytorch was used to create adequate filling and overhanging filling models (CranioCatch, Eskisehir, Turkey) in panoramic radiographs obtained from different orthopantomographic devices (Figure 1).

### Data Input

The data sets were obtained from the images of patients who applied to our clinic for various dental purposes. Radiographs were obtained using different panoramic devices (68–85 kVp, 10–14 mA, 10–13 s, minimum total beam filtering with a 2.5-mm Al equivalent layer and a pixel size of 48 μm). Images of individuals with mixed dentition were not included as this may cause errors during labeling. Radiographic images obtained by incorrect positioning of the patient or containing metal artifacts were excluded from the study. This study was conducted with 1850 images for overhanging fillings and 2473 images for adequate fillings.

### Labeling and Training of Data

Labeling is the process of identifying relevant regions in an image and deciding to which region they belong. For this purpose, the outer borders of the filling and overhanging fillings in the images were defined by polygonal segmentation and saved in the .JSON file type. Labeling was performed on panoramic radiography images by two restorative dentistry specialists on the same flat panel monitor in a semi-dark room. All labeling was then reviewed by three oral and maxillofacial radiology specialists. The collected data was analyzed using an artificial intelligence application. A total of 10480 labels were made in 2473 radiographs for filling, and 2491 labels were made in 1850 images for overhanging filling.

The segmentation model was first anonymized, then panoramic radiography images of varied sizes were resized to 640x320 resolution for dental filling and to 1280x640 resolution for overhanging tooth filling. A random sequence was created by using the open-source Python programming language and Opencv-Pytorch-Numpy-Pandas-Torch, Vision-Torch-Tensorboard-Seaborn libraries. To prevent the images participating in the training from being used for retesting, the data set was divided into three parts: 80% training, 10% validation, and 10% testing (Table 1).

**Table 1:** Distribution of training, testing, and validation groups for filling and overhanging filling.

Variable	Training Group	Testing Group	Validation Group
Filling	1978	247	248
Overfilling	1480	185	185

- Training group: The data used to train the model and makes up 80% of the dataset;
- Validation group: 10% of the data set that is independent of the training of the model and specifies the examples that the model should not see in this period. If it is necessary to end the training or revise the training variables, the model is tested on this data set;
- Test group: This group, which makes up 10% of the images, is the part where the model trained using the training and validation data is tested.

In order to estimate and generate the optimal AI algorithm weight factors, validation and training validation datasets were used. The model success was checked with the dataset of the test group that was trained using transfer learning with a pre-trained model.

### Deep-learning Algorithm

2D CNN architectures implemented using the PyTorch library in the training phase are very important for effective segmentation. YOLOv5, a state-of-the-art CNN architecture, plays a crucial role in training the model for dental filling and overhanging filling segmentation. 2D CNN architectures implemented with the PyTorch library in the Python programming environment were used. The model was trained using the YOLOv5x version segmentation algorithm over 500 epochs.

Deployment is also an essential requirement in real-life use. YOLOv5 uses a genetic algorithm to generate junction boxes. If the default ones are not good, the process is called auto anchor, which recalculates the junction boxes to fit the data. This is used in conjunction with the k-means algorithm to create advanced k-means anchor boxes. This is one of the reasons why YOLOv5 works so well, even on different datasets. Another reason for such good training and detection results of the YOLOv5 model is the mosaic amplification. In simple terms, mosaic amplification combines four different images into one image using different magnification techniques. This teaches the model to deal with different and complex images. The PyTorch model output was evaluated using a 10% portion of the test dataset, resulting in accuracy, precision, and recall metrics. Additionally, Mean Average Precision (mAP@0.5) values were computed, taking into account the IoU threshold set at 50%.

### Statistical Analysis

The performance of the model was evaluated using the data obtained in a confusion matrix. In statistical analysis in the context of artificial intelligence studies, the confusion matrix serves as a fundamental component for evaluating the performance of classification models. It provides a detailed comparison between the predicted and actual results (Table 1) enabling the assessment of classification accuracy and error patterns. The aim of using the confusion matrix is to systematically analyze the model’s ability to correctly classify instances into their respective classes and to identify areas for improvement. In addition to the confusion matrix, performance metrics such as true positive (TP), false positive (FP), and false negative (FN) values were also computed to gauge the model’s effectiveness.

Sensitivity (true positive rate, TPR), precision (positive predictive value, PPV), and F1 score were selected as key parameters for their relevance to the study’s objectives. By utilizing these metrics, statistical analysis aims not only to quantify the model’s performance but also to assess its robustness and generalization capabilities.

a. Sensitivity, true positive rate  (TPR): TP/ (TP + FN) 

b. Precision, positive predictive value  (PPV): TP/ (TP+FP)

2TP/(2TP+FP+FN)

## Results

**Figure 2:**
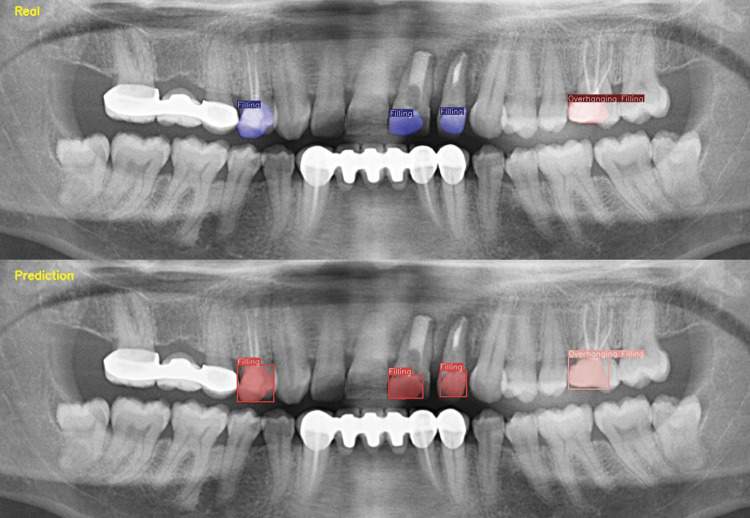
Estimated segmentation images generated by real and artificial intelligence model of overhanging filling and normal filling.

**Figure 3:**
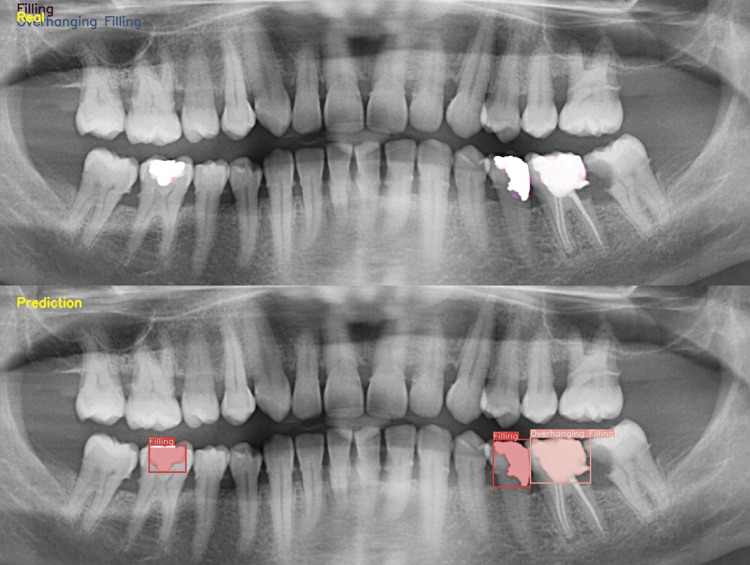
Panoramic radiograph depicting estimated segmentation images, like Figure 2, generated by a real and artificial intelligence model for detection of overhanging and normal fillings in a separate patient.

Within the scope of the study, 10480 labels were made in a total of 2473 images for filling, of which 8350 labels in 1978 images were used as the training dataset. For overhanging filling, a total of 2481 labels were made in 1850 images, and 1994 labels were used for 1480 images in the training dataset (Table 1). Sensitivity, precision, and F1 scores for filling were 0.95, 0.97, and 0.96, respectively. For overhanging filling, these values were 0.86, 0.89, and 0.87, respectively (Table 2). The model achieved a mAP@0.5 of 0.86 for the filling, while for the overhanging filling class, it demonstrated a mAP of 0.62. Additionally, the estimates of filling and overhanging filling restorations are illustrated in Figure 2 and 3.

**Table 2:** AI model prediction performance values from confusion matrix.

Variable	Sensitivity	Precision	F1 score
Filling	0.95	0.97	0.96
Overhanging filling	0.86	0.89	0.87

## Discussion

In the literature, diverse studies across various populations consistently report different rates of overhanging restorations, with an emphasis on their association with dental complications. The detection of carious lesions or overhanging restorations on the contact areas of posterior teeth can be challenging using conventional clinical examination methods. For this reason, a combination of clinical evaluations, such as visual and tactile examinations, and radiographic evaluations is the most reliable method for diagnosing overhanging margins. It is therefore recommended to use both clinical and radiographic evaluations when diagnosing overhanging margins.

In dentistry, intraoral and panoramic radiographs are common imaging techniques used to diagnose, treat and monitor patients. Although they provide 2D images of complex 3D structures, they have limitations such as lower image quality, geometric distortions, and overlaps that can affect the reliability of measurements. Standardization can also be challenging.^1,12^ Despite its lower resolution, panoramic radiography is a recommended method in dentistry for comprehensive diagnosis and treatment planning. This protocol enables the selection of additional intraoral periapical radiographs in specific areas, which can capture a larger area of the oral cavity while providing a complete examination of the teeth and surrounding bone with a lower radiation dose. The primary advantage of panoramic radiographs is that they show all teeth, which facilitates the detection of impacted teeth, foreign bodies in the jaws, and significant abnormalities in the number, position, and morphology of teeth.^13,14^ However, there is considerable variation in the ability of dentists to interpret panoramic radiographs, which can be influenced by their individual skills, experience, and biases. These limitations in the interpretation of panoramic radiographs may lead to misdiagnosis or inappropriate treatment.^14^

To overcome these challenges, deep learning (DL) models built on CNNs have recently emerged. They serve as the foundation for training computer vision systems. These models use various frameworks and methods, such as ResNet, Inception, Plain CNN, YOLO, and Detectron2, to perform tasks like classification, detection, and segmentation. By predicting an object’s bounding box, these frameworks can increase the accuracy of detection and segmentation tasks. YOLOv5 belongs to the category of computer vision models and is available in four primary versions: small (s), medium (m), large (l), and extra-large (x). These versions exhibit increasing levels of accuracy. YOLOv5 performs extremely well for this purpose compared to other innovative techniques. YOLOv5 makes training and inference on custom datasets extremely easy and relatively straightforward. It provides a quick training opportunity with a ready-made dataset in the proper format. Several export options are available, and completing an object detection pipeline involves more than just training and inference of models.

Our team conducted a study using the Faster R-CNN Inception v2 architecture, where we achieved an F1 score of 0.87 for the filling detection.^15^ Our primary aim was to investigate whether the YOLOv5x architecture, which is known for its success in the COCO dataset, can also improve the detection of filling in panoramic radiographs. By expanding our dataset and using a more advanced architecture, we observed a significant improvement in performance metrics. Since accurate and effective detection of restorations is a basic requirement for our study, the YOLOv5 algorithm was used, which performs effectively in terms of speed and accuracy, outperforming most of its previous versions. In the literature, the YOLOv5 model was later used in various applications, and the model began to gain confidence through effective results. In addition, YOLOv5’s real-time processing ability, ease of use, scalability, and adaptability to various fields and datasets, and its ability to stay up to date through constant feedback are other reasons for its use in our study.

Recent research has investigated the use of DL in a range of medical conditions and clinical situations, including tooth detection, localization, and identification of oral cancer. The potential of AI to improve treatment approaches in dentistry makes it a promising tool for future trends in healthcare thanks to its accuracy and versatility.^16^ Thanathornwong and Suebnukarn (2020) proposed the use of a DL-based object detection method to identify periodontally compromised teeth in digital panoramic radiographs.^13^ The aim was to reduce the effort required for diagnosis by saving evaluation time and providing automated screening documentation. The proposed approach has the potential to improve the precision and coherence of the diagnostic process while reducing the burden on clinicians. The broader potential of AI in dentistry, especially its accuracy and versatility, makes it a promising tool for future trends in healthcare.

Many studies have compared the performance of CNNs to that of human experts in various clinical scenarios. Despite potential inaccuracies in human examination methods, CNN methods have consistently demonstrated comparable accuracy, specificity, and sensitivity to human experts in various clinical scenarios.^10,17,18^ The study highlights the usefulness of digital technologies to improve dental practice and patient outcomes. There have been some studies on the application of deep learning algorithms for the radiographic detection of dental fillings in dental radiographs.

Baydar et al. (2023) developed a deep learning algorithm based on a convolutional neural network (CNN) to automatically detect dental fillings in bitewing radiographs. The CNN was trained on a dataset of over 1,000 bitewing radiographs and achieved an over 95% accuracy in detecting dental fillings.^19^ In another study with bitewing radiographs, researchers developed a deep learning algorithm based on a CNN to automatically detect overhanging fillings.^20^ Compared with our study findings, the success of the filling models was similar. Considering that bitewing radiographs provide more detailed images due to resolution and central beam angulation, we think that the success of the models for panoramic radiographs is high and that it will increase with the use of the new CNN model and the participation of diversified labels in a large amount of data.

Besides, these studies used deep convolutional neural networks (DCNNs) as the CNN architecture for their deep learning models. The specific models used were VGG-16/19, AlexNet, and ResNet-50. VGG-16 and ResNet-50 are both well-established and widely used CNN architectures with a large number of pre-trained models available. This can make it easier to implement and fine-tune models for specific tasks. They have been shown to perform well on a wide range of image classification tasks, including medical imaging tasks. These architectures can be easily trained on relatively small datasets. However, both VGG-16 and ResNet-50 are relatively deep models with a large number of parameters, which can make them slower to train and require more computational resources. These architect models are primarily designed for image classification tasks and may not perform as well on object detection tasks, such as identifying the exact location of dental fillings. YOLOv5 is specifically designed for object detection tasks, which makes it well-suited for identifying the exact location of normal fillings or overhanging fillings in radiographs. YOLOv5 has a relatively light architecture compared to VGG-16 and ResNet-50, which means it can be trained faster and require fewer computational resources.^21-23^

In their 2021 study, Mao et al. investigated four distinct CNN models for restoration detection, evaluating their performance and establishing GoogleNet as the most effective.^16^ Notably, Mao et al.’s study lacked categorization of restorations based on factors such as normal/overhanging, a distinction that our current study incorporates. Furthermore, their comparison of models did not include the Yolo model, which we have employed in our study. Çelik and Çelik (2022) investigated the success of 10 different CNN algorithms, including Yolo-v3, in restoration and implant identification, and found the success of YOLO to be lower. However, in that study, the researchers preferred object detection for labeling. Since we used the segmentation method for labeling in our study, we benefited from the success of YOLO in this sense.^24^

To the best of our knowledge, there is no study that investigated the success of model for detecting overhanging filling, and the success of a YOLO-v5x model developed in this study has been presented. While the impact of dental fillings on segmentation success in both 2- and 3-dimensional tooth segmentation remains a topic of debate, no studies have evaluated the effectiveness of DL algorithms, the most commonly utilized method in dentistry, for detecting dental fillings. It is worth noting that comparing detection performance across studies may be unrealistic, particularly considering that structures labelled in other studies predominantly exhibit radiolucent characteristics, such as caries and apical lesions, falling within the scope of density-based analysis. The use of deep learning algorithms for detecting normal fillings and overhanging fillings on panoramic radiographs shows promise for improving the accuracy and efficiency of dental diagnosis and treatment planning. In addition, a noteworthy strength of our research lies in the images from different devices. This multi-device approach not only broadened the scope of our analysis but also enhanced the robustness and generalizability of our findings. Incorporating data from various imaging platforms allowed us to account for potential device-specific variations, providing a more comprehensive understanding of the phenomena under investigation. This diversity in image sources contributes with the reliability and robustness of our findings, reflecting the real-world scenario where a variety of imaging devices are used by dentists. This approach sets our research apart and establishes a solid foundation for future investigations in this field.

A limitation of our study is that it relies exclusively on retrospective radiology without incorporating clinical dental examinations, which are fundamental for a comprehensive dental assessment. However, our study indirectly aims to expedite the identification of areas that should be carefully examined during intra-oral evaluations, rather than completely disregarding the clinical assessment. This approach should make targeted treatment planning more effective. Another limitation is the exclusion of radiographs with errors/poor image quality that occur in the reality of the dental clinical workflow. This situation partially increases the success of the model and distances it relatively far from the reality of dental practice. Although only one algorithm was used in our study, which has the advantage of today’s simple and fast application and a high success rate, future research should compare multiple algorithms in the field of data engineering and AI. Moreover, longitudinal studies evaluating the long-term impact of DL on treatment planning are essential to understand the full potential of artificial intelligence in dentistry. These avenues of investigation are likely to provide more robust findings regarding the feasibility and effectiveness of artificial intelligence applications in dental practice.

## Conclusion

In conclusion, our study developed a YOLOv5-based DL algorithm, achieving high accuracy with a 0.95 sensitivity, 0.97 precision, and F1 score of 0.96 for normal filling, and a 0.86 sensitivity, 0.89 precision, and F1 score of 0.87 for overhanging filling. The results highlight the proficiency of YOLOv5 in accurately identifying dental anomalies, offering promising applications for enhanced efficiency and precision in dental diagnosis and treatment planning. While acknowledging the study successes, future research should address the inherent limitations, ensuring the algorithm’s robustness across diverse clinical scenarios. The YOLOv5 algorithm emerges as a valuable tool that will transform dental diagnostics, promising improved accuracy and efficiency in the identification of normal fillings and overhanging fillings.
